# Nervonic acid amends motor disorder in a mouse model of Parkinson’s disease

**DOI:** 10.1515/tnsci-2022-0450

**Published:** 2022-04-20

**Authors:** Dandong Hu, Yujuan Cui, Ji Zhang

**Affiliations:** School of Life Science, Northwest Normal University, Lanzhou, Gansu 730070, People’s Republic of China; Beijing Yanqing District Food and Drug Safety Monitoring Center, Beijing Yanqing Center for Diseases Prevention and Control, Beijing, 102100, People’s Republic of China

The publisher would like to inform that the article “Nervonic acid amends motor disorder in a mouse model of Parkinson’s disease” by Dandong Hu, Yujuan Cui, and Ji Zhang (https://doi.org/10.1515/tnsci-2020-0171) has been retracted.

The authors simultaneously submitted and published the manuscript in two journals. Although the article was first accepted in Translational Neuroscience, the Editorial Board decided to retract the publication due to a serious violation of publishing ethics. A duplicated article was published in the Neurochemical Journal (Pleiades Publishing).

